# Extended high-frequency audiometry: hearing thresholds in adults

**DOI:** 10.1007/s00405-022-07498-1

**Published:** 2022-06-28

**Authors:** Michaela Škerková, Martina Kovalová, Tomáš Rychlý, Hana Tomášková, Hana Šlachtová, Zdeněk Čada, Rastislav Maďar, Eva Mrázková

**Affiliations:** 1 Department of Epidemiology and Public Health, Faculty of Medicine, University of Ostrava, 703 00 Ostrava, Czech Republic; 2 Hospital Center for Hearing and Balance Disorders, 708 00 Ostrava, Czech Republic; 3 Department of Otorhinolaryngology and Head and Neck Surgery, 1st Faculty of Medicine Charles University in Prague and Motol University Hospital, Postgraduate Medical School, 150 06 Prague 5, Czech Republic; 4 Department of ENT, Regional Hospital Havirov, 736 01 Havirov, Czech Republic

**Keywords:** Hearing loss, Audiometry, Hearing test, Extended high-frequency audiometry, Hearing threshold, Audiogram

## Abstract

**Purpose:**

This study aimed to determine hearing thresholds in an otologically normal population without occupational noise exposure aged 18 to 64 years using extended high-frequency audiometry (EHFA).

**Methods:**

Individuals from the general population who have never had hearing problems and whose job was not associated with noise exposure were included in the study and classified by age into 5 categories: 18–24 and, further, by 10 years of age. Each of these groups was further divided according to gender. All subjects underwent tympanometry, conventional pure-tone audiometry within the 0.125–8 kHz range, and extended high-frequency audiometry within the 9–16 kHz range, performed according to the standards. The significance level for statistical testing was set at 5%.

**Results:**

Here, we established hearing thresholds in an otologically healthy population within the extended high-frequency (EHF) range (9–16 kHz). We found the EHFA to be a highly sensitive method for early detection of hearing loss, with hearing thresholds decreasing as soon as 35 years of age. In males, the hearing thresholds grew with age more rapidly than in women. The ability to respond at EHF gradually decreased with age and increasing frequency.

**Conclusion:**

Our results can help improve the knowledge of EHF hearing thresholds for individual sexes and age groups. So far, the standard 7029:2017 is not binding and, moreover, it only reaches up to the frequency of 12.5 kHz. EHFA is a highly sensitive method for the evaluation of hearing loss depending on age and sex.

## Introduction

Hearing impairment is globally a highly topical issue. Nearly 2.5 billion people worldwide will be living with some degree of hearing loss by 2050, warns the World Health Organization’s (WHO) first World Report on Hearing. At least 700 million of these people will require access to ear and hearing care and other rehabilitation services unless action is taken [1]. Hearing impairment, if not identified and addressed, can have far-reaching consequences, adversely affecting language development, psychosocial well-being, quality of life, educational attainment, and economic independence at various stages of life [2, 3]. Unaddressed, hearing loss imposes a global annual cost of more than $980 billion. Causes of hearing impairment and hearing loss are multifactorial, including genetic causes, complications at birth, infectious diseases, the use of ototoxic medications, sex, exposure to noise, aging, etc. Among this variety of factors involved in hearing loss, aging is one of the most widely recognized [2, 4]. Hearing loss also comes with consequences, for example, it has been proven to be the most significant risk factor for dementia development in middle-aged people (45–65 years of age) [5]. Hearing impairment should be revealed as soon as possible; this is, however, typically not the case as adults typically underestimate the seriousness and implications of any hearing problems, thus delaying treatment. Hearing aids are, therefore, predominantly reaching the older population. The COVID-19 pandemic has underlined the importance of hearing. As we have struggled to maintain social contact and remain connected to family, friends, and colleagues, we have relied on being able to hear them more than ever before. Covering mouths with face masks made lip-reading also impossible, which made many people with impaired hearing who might have not even been aware of this problem start to take interest in their hearing [2]. Preventing and treating diseases and disabilities of all kinds should not be perceived as a cost but rather as an investment in a safer, fairer, and more prosperous world for all people [2]. As early diagnosis is the key to the treatment of practically any disease, finding a method capable of diagnosing hearing disorders at an early stage is of great importance. Extended high-frequency (EHFA), a method used to examine hearing thresholds in the frequency range of 8–20 kHz, could be valuable in this context as the damage to hearing thresholds can typically be first observed at these frequencies [6]. EHFA is, therefore, a very useful test, which can detect hearing loss early, i.e., before it starts involving the medium and low frequencies that significantly affect hearing capacity [7]. EHFA has been studied for several decades but the lack of commercially available equipment (adapted audiometers capable of generating tones with frequencies of up to 20 kHz are used for the test) and the standardization of calibration recommendations have been limiting its use for a long time [8]. The EN ISO 7029 standard valid for frequencies of 0.125–8 kHz in individuals with normal hearing was under development for decades [9]. The current version, EN ISO 7029:2017, remains only informative for hearing thresholds at frequencies from 9 to 12.5 kHz and no hearing thresholds have been established for higher frequencies [9]. The presented study aimed to determine hearing thresholds at high frequencies (9–16 kHz) in an otologically normal population aged 18–64 without professional noise exposure using EHFA.

## Methods

### Study population

This study was performed in accordance with the Declaration of Helsinki and approved by the Ethics Committee of the University of Ostrava. All individuals completed and signed an informed consent form prior to inclusion in the study. The data were collected between 2020 and 2021 at the ENT outpatient clinic, which was a part of a multidisciplinary facility. Only individuals who have never had any hearing problems, self-assessed their hearing as “normal” and neither otoscopy nor tympanometry revealed any abnormalities were included in the study. The study group consisted of individuals aged 18–64 years who worked in a job without professional noise exposure (A-weighted equivalent sound pressure level L_Aeq,8 h_ < 80 dB), randomly selected from the database of the facility who were not previously patients of the ENT clinic were offered participation. The study group was drawn from the socially consistent general population living in the same industrial region burdened also with traffic noise. Statistical evaluation was then limited only to data from participants whose tympanic membrane was assessed to be normal during otoscopic examination, whose immittance test of the middle ear (type A tympanometry curve) was normal and hearing loss at frequencies of 0.5; 1; 2; and 4 kHz was lower than 25 dB. The history of noise exposure in public areas and leisure activities was not investigated in this work; however, professional exposure to workplace noise was an exclusion criterion. The recruited individuals were divided into age categories of 18–24, 25–34, 35–44, 45–54, 55–64 years. The exclusion criteria were as follows: disagreement with inclusion in the study or with signing informed consent, age outside the range of 18–64 years, professional noise exposure, pathological result of an otoscopic examination, type B or C tympanometric curve, or hearing threshold of more than 25 dB at 0.5, 1, 2, or 4 kHz.

### Hearing measurement

Participants had been advised in the invitation that 24 h before the examination, they should not use personal audio devices and they should avoid exposure to excessive noise. Before beginning the examination, the participants were briefed about the process and the principle of the audiometric measurements. Data collection began with taking a brief personal history and other data needed for further processing. This was followed by tympanometry (Madsen Zodiac Diagnostic, type 1096), conventional pure-tone audiometry, and high-frequency audiometry (Madsen Astera 2, Headset Sennheiser HDA300), performed according to the standards EN ISO 8253-1:2010 Acoustics—Audiometric test methods and EN ISO 266:1997 Acoustics—Preferred frequencies [10, 11]. These standards were last reviewed and confirmed in 2018 and 2021, respectively; as such, these versions can be considered current. All instruments were calibrated before the beginning of the measurement. The examination was performed always by the same personnel in an acoustic chamber. The respondents were equipped with headphones, through which tones of different intensities and frequencies were played, first into one ear, then into the other. The measurement started with the ear that the participant identified as the one with better hearing. If the participant did not perceive a subjective difference of hearing between the ears, the measurement began in the left ear. The hearing threshold was measured at conventional (0.125, 0.25, 0.5, 0.75, 1, 1.5, 2, 3, 4, 6, 8 kHz), as well as extended high frequencies (EHF; 9, 10, 11.25, 12.5, 14, and 16 kHz). The result was plotted as an audiogram in which the hearing thresholds was expressed in dB (decibel hearing level, dB HL) for each frequency and separately for each ear. The average values of hearing loss for individual frequencies were calculated, the average threshold curves for individual age categories were compiled and statistically compared to determine the differences in hearing thresholds (in dB) among groups.

### Statistical analyses

The study results were exported to Microsoft Office Excel 2017 (MS Excel; Microsoft Corporation, Washington, DC, USA) for calculation of basic descriptive statistics and to create tables and graphs. Data were analyzed using basic descriptive statistics, Pearson’s chi-squared test and the nonparametric Mann–Whitney *U* test. Statistical significance was analyzed using the Stata version 13 software (Data Analysis and Statistical Software; StataCorp LP, CollegeStation, TX, USA). The significance level for testing was set at 5%.

## Results

### Study population

In all, 316 participants (i.e., 632 ears) aged 18–64 were included in the study, of which 68% were women and 32% men, respectively. There was no difference in the sex distribution among age groups (*p* = 0.928; Table 1).

**Table 1:** Number of ears and the percentage representation of men and women in individual age categories

Age groups	18–24	25–34	35–44	45–54	55–64	Total
Men's ears	32 (15.8%)	62 (30.7%)	30 (14.9%)	38 (18.8%)	40 (19.8%)	202 (100%)
Women's ears	76 (17.7%)	124 (28.8%)	78 (18.1%)	76 (17.7%)	76 (17.7%)	430 (100%)
Total ears	108 (17.1%)	186(29.4%)	108 (17.1%)	114 (18.0%)	116 (18.4%)	632 (100%)

*Chi-squared test; data are presented as *N* (% in the respective age group)

**Fig. 1:**
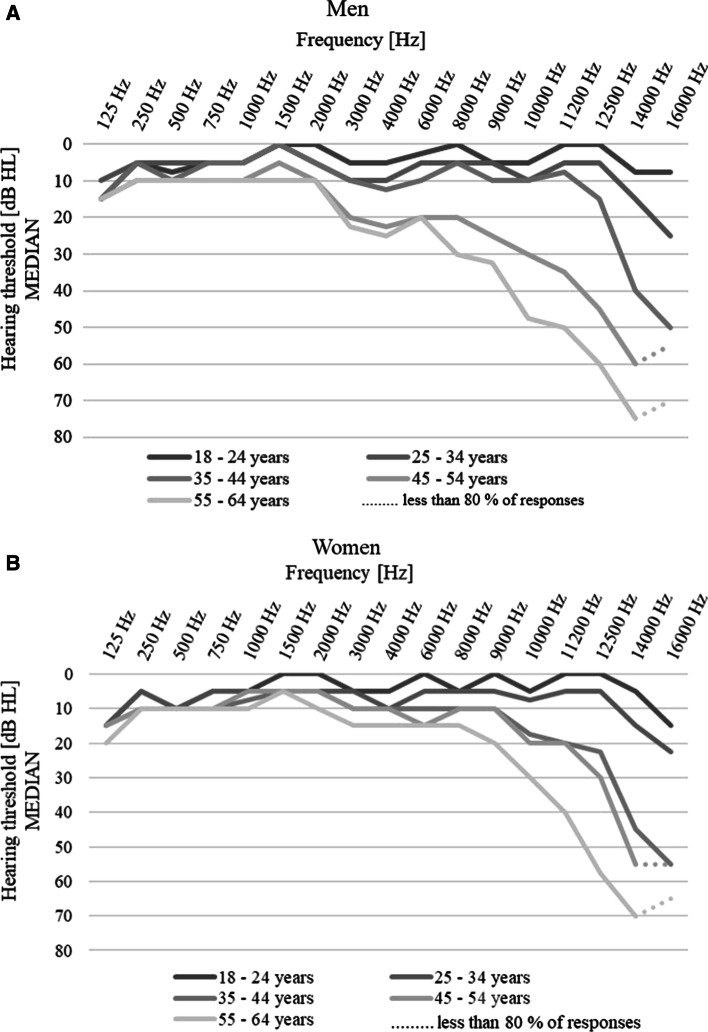
Median hearing thresholds for individual ages and frequencies in men (**A**) and women (**B**)

The average threshold curves for individual age categories in men and women are shown in Fig. 1A, B. It is obvious that with increasing age, a gradual deterioration of the hearing threshold, especially at high frequencies, is observed in both sexes. Compared to women, the increase in hearing thresholds was more pronounced in men aged 45–54 and 54–64 years for both conventional as well as high frequencies.

### Comparison of left and right ears

A statistical analysis of hearing thresholds between the right and left ears for each sex and age group was performed. No statistically significant difference was found and for this reason, further analyses do not distinguish the laterality.

### Comparison of hearing threshold medians between sexes

Table 2 details median hearing thresholds at frequencies 0.125–16 kHz in men and women. The hearing thresholds generally increased with age and frequency; this observation was more pronounced in men. The biggest differences in hearing thresholds between sexes were observed in the 35–44 years age group at the high frequency of 11.25 kHz (12.5 dB), 45–54 years age group at 9, 11.25, and 12.5 kHz (15 dB), 55–64 years at 8 kHz (15 dB), 9 kHz (12.5 dB), and 10 kHz (17.5 dB). In the younger age groups below 44 years of age, the differences did not exceed 5 dB (except for frequencies of 11.25 and 12.5 kHz in the age groups of 35–44 years).

**Table 2:** Hearing thresholds and significant differences by age group and sex

	Hearing threshold [dB] in medians (Q1;Q2)									
	Men (total 202 ears)					Women (total 430 ears)				
Number of ears	32	62	30	38	40	76	124	78	76	76
Age groups	18–24 years	25–34 years	35–44 years	45–54 years	55–64 years	18–24 years	25–34 years	35–44 years	45–54 years	55–64 years
*Frequency [kHz]*										
0.125	15 (10;15)	10 (10;15)	15 (10;15)	15 (15;20) ⁑●	15 (13.75;20) ⁑●	15 (10;15)	15 (10;15)	15 (10;20) ⁑●	15 (10;20) ⁑●	20 (15;21.25) ⁑●▲
0.25	5 (0;10)	5 (5;10)	♀ 5 (5;10)	10 (5;15) ⁑●▲	10 (5;15) ⁑●▲	5 (5;10)	5 (3.75;10)	10 (5;10) ⁑●	10 (5;15) ⁑●	10 (8.75;15) ⁑●▲○
0.5	7,5 (5;10)	♀ 5 (5;10)	♀ 10 (6.25;10)	10 (5;18.75) ⁑●	10 (10;20) ⁑●▲	10 (5;10)	10 (5;11.25) ⁑	10 (10;15) ⁑●	10 (5;15) ⁑●	10 (10;15) ⁑●
0.75	5 (0;10)	5 (5;10)	♀ 5 (5;10)	10 (5;15) ⁑●▲	10 (5;16.25) ⁑●▲	5 (0;10)	5 (5;10) ⁑	10 (5;15) ⁑●	10 (5;15) ⁑●	10 (8.75;15) ⁑●
1	5 (0;6.25)	♀ 5 (0;5)	♀ 5 (5;10) ●	10 (5;10) ⁑●▲	10 (5;20) ⁑●▲	5 (0;5)	5 (5;10) ⁑	7.5 (5;10) ⁑●	5 (5;10) ⁑●	10 (5;15) ⁑●▲○
1.5	0 (0;5)	♀ 0 (0;5)	♂ 0 (0;5)	5 (2.5;10) ⁑●▲	10 (5;15) ⁑●▲	0 (0;5)	5 (0;5)	15 (0;10) ⁑	5 (0;10) ⁑	5 (5;10) ⁑●
2	0 (0;5)	5 (0;5)	♂ 5 (0;5)	10 (5;15) ⁑●▲	♂ 10 (5;20) ⁑●▲	0 (0;5)	5 (0;10) ⁑	5 (5;15) ⁑●	5 (5;10) ⁑●	10 (5;15) ⁑●
3	5 (3.75;10)	10 (5;10)	10 (5;15) ⁑	♂ 20 (15;25) ⁑●▲	♂ 22.5 (15;35) ⁑●▲	5 (0;10)	5 (5;10)	10 (10;15) ⁑●	10 (5;15) ⁑●	15 (10;20) ⁑●▲○
4	5 (0;5)	12,5 (5;13.75) ⁑	10 (5;20) ⁑●	♂ 22.5 (20;35) ⁑●▲	♂ 25 (15;36.25) ⁑●▲	5 (0; 10)	10 (5;10) ⁑	10 (6.25;15) ⁑●	10 (5;20) ⁑●	15 (10;20) ⁑●▲○
6	2.5 (0;10)	10 (5;10) ⁑	7.5 (5;15) ⁑	♂ 20 (15;33.75) ⁑●▲	♂ 20 (20;35) ⁑●▲	0 (0;5)	5 (0;10) ⁑	10 (5;15) ⁑●	15 (10;20) ⁑●▲	15 (10;25) ⁑●▲
8	0 (0;5)	5 (0;8.75)	5 (0;10) ⁑●	♂ 20 (10;28.75) ⁑●▲	♂ 30 (13.75;40) ⁑●▲○	5 (0;10)	5 (0;10)	10 (5;15) ⁑●	10 (5;16.25) ⁑●	15 (10;25) ⁑●▲○
9	♂ 5 (0;10)	10 (0;5)	7.5 (5;10) ●	♂ 25 (10;35) ⁑●▲	♂ 32.5 (20;51.25) ⁑●▲○	0 (0;5)	5 (0;10) ⁑	10 (5;20) ⁑●	10 (5;25) ⁑●	20 (10;36.25) ⁑●▲○
10	5 (0;15)	10 (5;15)	12.5 (5;15) ⁑	♂ 30 (15;47.5) ⁑●▲	♂ 47.5 (33.75;61.25) ⁑●▲○	5 (0;10)	7.5 (5;10) ⁑	17.5 (10;28.75) ⁑●	20 (10;30) ⁑●	30 (20;55) ⁑●▲○
11.25	0 (0;10)	7.5 (0;10)	♀ 7.5 (0;15) ⁑●	♂ 35 (16.25;55) ⁑●▲	50 (38.75;61.25) ⁑●▲○	0 (0;5)	5 (0;10) ⁑	20 (6.25;33.75) ⁑●	20 (10;40) ⁑●	40 (30;60) ⁑●▲○
12.5	0 (0;10)	5 (0;15)	♀ 15 (5; 28.75) ⁑●	♂ 45 (26.25;65) ⁑●▲	60 (55;75) ⁑●▲○	0 (0;6.25)	5 (0;10) ⁑	22.5 (10;45) ⁑●	30 (15;46.25) ⁑●	57.5 (40;70) ⁑●▲○
14	7.5 (0;21.25)	15 (10;25)	40 (25; 48.75) ⁑●	60 (46.25;73.75) ⁑●▲	75 (65;85) ⁑●▲○	5 (0;15)	15 (5;25) ⁑	45 (30;63.75) ⁑●	55 (45;65) ⁑●	70 (65;80) ⁑●▲○
16	7.5 (0;20)	25 (15;30) ⁑	50 (40; 58.75) ⁑●	55 (55;65) ⁑●▲	70 (55;70) ⁑●▲○	15 (0;25)	22.5 (15;40) ⁑	55 (45;60) ⁑●	55 (45;60) ⁑●	65 (55;70) ⁑●▲○

Q1 25th empirical quartile; Q2 75th empirical quartile

⁑Significant difference (*p* < 0.05) from the group of the 18–24 years; ● significant difference (*p* < 0.05) from the group of the 25–34 years; ▲ significant difference (*p* < 0.05) from the group of the 35–44 years; ○ significant difference (*p* < 0.05) from the group of the 45–54 years

♀ significant difference (*p* < 0.05) between sex and the age same group (higher hearing threshold in women); ♂ significant difference (*p* < 0.05) between sex and the age same group (higher hearing threshold in men)

### Comparison of median hearing thresholds among age groups

The general trends for men and women were similar both for conventional and high frequencies (see Table 2). In men, there were generally no differences in hearing thresholds in the two youngest age groups; after that, however (i.e., from the age group of 35–44 onwards), hearing thresholds in all age groups mutually differed, with hearing thresholds increasing with increasing age. In women, no statistically significant differences were observed between the age groups of 35–44 and 45–54; however, apart from these, all other groups (in general) mutually differed, with thresholds increasing with increasing age. Hearing loss of > 25 dB was observed at some high frequencies in the age groups 35–44, 45–54 and 55–64. In men over 45 years of age, hearing losses of > 25 dB were observed at all EHF frequencies with the exception of 9 kHz; in the 55 + years group, this included the 9 kHz as well. In women, the hearing loss > 25 dB was recorded in women of 35 years and older, especially at frequencies of 14 and 16 kHz. In the 55–64 age group, hearing thresholds were increased at all EHF. The ability to respond in % in all measured frequencies by age groups is shown in Table 3.

**Table 3:** The ability to respond (%) in all measured frequencies by age groups

	Men					Women				
Frequency [kHz]	32 ears	62 ears	30 ears	38 ears	40 ears	76 ears	124 ears	78 ears	76 ears	76 ears
	18–24 years	25–34 years	35–44 years	45–54 years	55–64 years	18–24 years	25–34 years	35–44 years	45–54 years	55–64 years
0.125–11.2	100%	100%	100%	100%	100%	100%	100%	100%	100%	100%
12.5	100%	100%	100%	100%	98%	100%	100%	99%	99%	99%
14	100%	100%	100%	97%	85%	100%	100%	97%	96%	93%
16	100%	98%	97%	79%	48%	99%	100%	87%	75%	53%

## Discussion

The presented study aimed to establish normal hearing levels at extended high frequencies in an otologically healthy population of 5 age categories (18–64) for both sexes. The results of this work can significantly contribute to the current knowledge at EHFas at present, the standard EN ISO 7029:2017 has only informative value for this range with a maximum of 12.5 kHz [9]. As the standard gradually develops taking into account new studies, the presented one has the ambition to contribute to establishing the normative values of hearing thresholds at extended high frequencies. Previously published studies monitoring high-frequency hearing loss in individuals professionally unexposed to noise reported an increase in hearing thresholds with an increasing frequency as well as increasing age, while the ability to respond to higher frequencies declined with age [12–17], which is in accordance with our study. Hearing thresholds at the extended high frequencies (9 to 20 kHz) are more sensitive to aging than frequencies of up to 8 kHz [16, 18].

Our results imply that it is important to distinguish between sexes but not between the right and left ears as the results for both sides are similar. This is in agreement with the study by Barbosa de Sá who reported that the thresholds were similar in the left ear and right ear, with significant differences between the ears only being observed at 11 kHz and 12 kHz, at which the right ear performed worse [12]. Another study, however, reported different hearing thresholds between ears in the same individuals, with the right ear performing generally worse [14].

Valiente et al. analyzed a group aged 5–90 with groups of 5–19 and, afterward, groups of 10 years of age. The mean hearing thresholds at each frequency (11.2 to 20 kHz) were lower in women than in men [15]. This agrees with our results as in our study, in which this difference has been observed as soon as in the youngest age category. However, other studies showed that the hearing thresholds for men and women were similar [12, 16]. Barbosa et al. analyzed high-frequency hearing thresholds in individuals aged 18–29 years and found no significant differences in hearing thresholds between men and women [12]. This, however, also agrees with our study as in the age category of 18–25 years, we found no significant differences in hearing thresholds between sexes, either. Similarly, Wang et al. did not find any significant difference in hearing thresholds between sexes within each age group at most of the frequencies from 0.25 to 20 kHz, (differences between men and women were detected only at frequencies of 4, 6, 8, and 9 kHz in the 41–50 year group) [16]. This could, however, be associated with a relatively smaller number of participants in their study (162) compared to ours, which might have resulted in insufficient power to detect any differences between sexes. Besides, the differences in hearing thresholds between sexes might have been masked due to multifactorial causes, including, e.g., smoking or abnormal BMI.

Wang et al. showed that hearing at EHF started to degenerate in subjects at their 30 s and the degeneration intensified in their 50 s and older age. The hearing thresholds of EHF were less than 26 dB before 30 years of age; with aging, however, the mean threshold values increased up to 75 dB [16]. In our group, the hearing thresholds of EHF in both sexes were higher than 26 dB in the age categories from 35 years onwards; this was initially observed only at the highest frequencies (14 and 16 kHz) but with increasing age, this extended to the lower frequencies as well. In men, we registered a notable deterioration of hearing in the 45–54 years age group at all EHF. In women, this was observed, especially at frequencies of 12.5, 14, and 16 kHz, as soon as in the 35–44 years category.

The ability to respond at EHF gradually decreased with age and increasing frequency up to 48% and 53% at 16 kHz in men and women in the age category of 55–64 years, respectively. However, at the frequency of 12 kHz, this parameter remained high even at the highest age group (99% women and 98% men). Our results indicate a higher ability to respond to EHF than the aforementioned Chinese [16] and Spanish [15] studies. When looking for explanations, we can also consider the environmental settings (air pollution, smoking, exposure to background noise, etc.). The poorer ability to respond might have been caused in those studies, among other things, also by older instrumentation or, as suggested above, long-term exposure to different levels of background noise; while our study was performed in an agglomeration of about 500,000 population, Valiente et al. performed the study in Madrid with 3.5 million population and Wang in Jinan with about 9.2 million population. In both those agglomerations, the long-term background noise exposure is likely much higher than in our study area [15, 16].

Only a few published studies have used EHF to determine hearing thresholds in a professionally unexposed population. The comparison between studies is, however, often difficult due to differences in age groups, sex representation, evaluation of risk factors, or, possibly, used audiometers and their accessories (papers originate from 2001 to 2021) [19, 20]. Study limitations include relatively small numbers of individuals in individual age/sex groups. As otitis media can also affect hearing [21], the fact that we did not collect anamnestic data about otitis in the history of individual patients can also be considered a limitation to our study.

## Conclusion

In this study, hearing thresholds at various frequencies (both conventional and extended high frequencies), age groups, and in both sexes were measured in a healthy population to propose normal hearing thresholds in the Central European population. In addition, we found that EHFA is a highly sensitive method for early capture of hearing loss. Hearing thresholds begin to deteriorate since 35 years of age; for this reason, individuals with a higher risk of hearing loss should be diagnosed at such an early age. In our study, we confirmed the differences in hearing thresholds between men and women while the differences between the right and left ears were statistically insignificant. In men, the hearing thresholds grew significantly faster than in women.

## Data Availability

The raw datasets generated and analyzed during the current study are available from the corresponding author on reasonable request.

## Abbreviations

EHF: Extended high-frequency
EHFA: Extended high-frequency audiometry
WHO: World Health Organization
